# Analysis of impulse oscillometric measures of lung function and respiratory system model parameters in small airway-impaired and healthy children over a 2-year period

**DOI:** 10.1186/1475-925X-10-43

**Published:** 2011-06-01

**Authors:** Erika G Meraz, Homer Nazeran, Carlos D Ramos, Pat Nava, Bill Diong, Michael D Goldman, Christine A Goldman

**Affiliations:** 1 Department of Electrical and Computer Engineering, The University of Texas at El Paso, El Paso, Texas, USA; 2 Universidad Autónoma de Ciudad Juárez, Chihuahua, México; 3 Department of Engineering, Texas Christian University, Fort Worth, Texas, USA; 4 Geffen School of Medicine, University of California at Los Angeles, California, USA; 5 Veterans Administration Medical Center, Los Angeles, USA

## 

After the publication of this work [1], we became aware of the fact that one author was missing on the author list. Christine A Goldman collaborated closely in quality assuring the IOS data and contributed to the design and simplification of graphs representing and illustrating the parameter expressed as AX, the Goldman Triangle.
